# 2-Nitro­benzyl methane­sulfonate

**DOI:** 10.1107/S160053681400899X

**Published:** 2014-04-30

**Authors:** Venkatramu Anuradha, S. Madan Kumar, B. P. Siddaraju, N. K. Lokanath, P. Nagendra

**Affiliations:** a Department of Physics, Dr M. G. R. Educational and Research Institute, Maduravoyal, Chennai, India; b Department of Studies in Physics, University of Mysore, Manasagangotri, Mysore 570 006, India; c Department of Chemistry, BET Academy of Higher Education, Bharathi College, Bharthi Nagara, Mandya 571 422, India

## Abstract

In the title compound, C_8_H_9_NO_5_S, the dihedral angle between the benzene ring and the nitro group is 5.86 (15)° and the C—C—O—S group adopts an *anti* conformation [torsion angle = −168.44 (15)°]. In the crystal, mol­ecules are linked by C—H⋯O hydrogen bonds, generating a three-dimensional network.

## Related literature   

For background to nitro­benzene derivatives, see: Ranu & Banerjee (2005 ▶); Ballini *et al.* (2005 ▶). For a related structure, see: Khan *et al.* (2008 ▶).
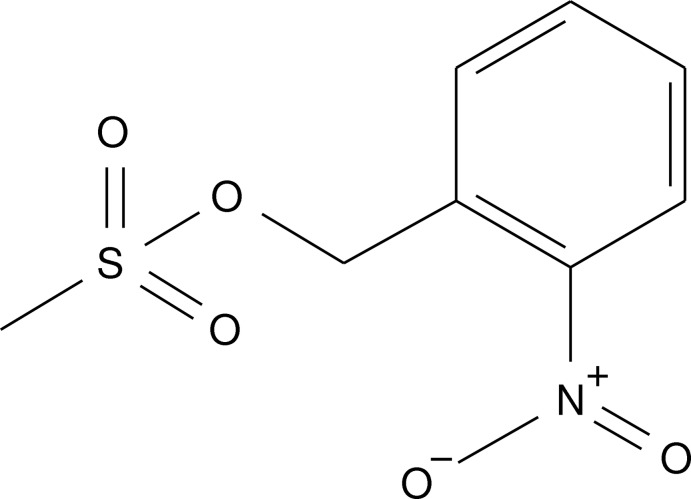


## Experimental   

### 

#### Crystal data  

C_8_H_9_NO_5_S
*M* *_r_* = 231.23
Monoclinic, 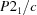

*a* = 12.414 (3) Å
*b* = 7.967 (2) Å
*c* = 10.994 (3) Å
β = 112.235 (11)°
*V* = 1006.5 (5) Å^3^
*Z* = 4
Cu *K*α radiation
μ = 2.94 mm^−1^
*T* = 296 K
0.23 × 0.22 × 0.21 mm

#### Data collection  

Bruker X8 Proteum CCD diffractometer
Absorption correction: multi-scan (*SADABS*; Bruker, 2013 ▶) *T*_min_ = 0.552, *T*_max_ = 0.578
6524 measured reflections
1663 independent reflections
1541 reflections with *I* > 2σ(*I*)
*R*_int_ = 0.047

#### Refinement  

*R*[*F* ^2^ > 2σ(*F* ^2^)] = 0.045
*wR*(*F* ^2^) = 0.112
*S* = 1.08
1663 reflections
138 parameters
H-atom parameters constrained
Δρ_max_ = 0.41 e Å^−3^
Δρ_min_ = −0.32 e Å^−3^

### 

Data collection: *APEX2* (Bruker, 2013 ▶); cell refinement: *SAINT* (Bruker, 2013 ▶); data reduction: *SAINT*; program(s) used to solve structure: *SHELXS97* (Sheldrick, 2008 ▶); program(s) used to refine structure: *SHELXL97* (Sheldrick, 2008 ▶); molecular graphics: *Mercury* (Macrae *et al.*, 2008 ▶); software used to prepare material for publication: *SHELXL97*.

## Supplementary Material

Crystal structure: contains datablock(s) global, I. DOI: 10.1107/S160053681400899X/hb7217sup1.cif

Structure factors: contains datablock(s) I. DOI: 10.1107/S160053681400899X/hb7217Isup2.hkl

Supporting information file. DOI: 10.1107/S160053681400899X/hb7217Isup3.cml

CCDC reference: 998614

Additional supporting information:  crystallographic information; 3D view; checkCIF report

## Figures and Tables

**Table 1:** Hydrogen-bond geometry (Å, °)

*D*—H⋯*A*	*D*—H	H⋯*A*	*D*⋯*A*	*D*—H⋯*A*
C2—H2⋯O1^i^	0.93	2.54	3.266 (3)	135
C3—H3⋯O2^ii^	0.93	2.53	3.335 (3)	145
C7—H7*B*⋯O4^iii^	0.97	2.58	3.539 (3)	169
C8—H8*A*⋯O4^iii^	0.96	2.42	3.374 (4)	172

Symmetry codes: (i) 
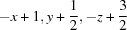
; (ii) 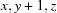; (iii) 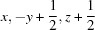.

**Fig. 1.**
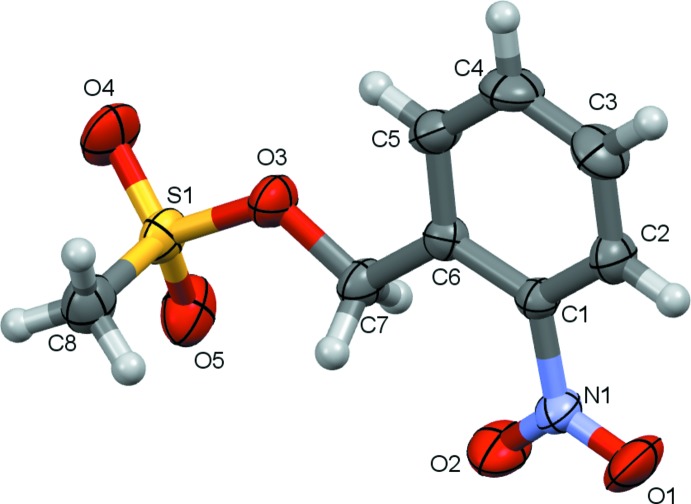
A view of the title molecule, with displacement ellipsoids drawn at the 50% probability level.

**Fig. 2.**
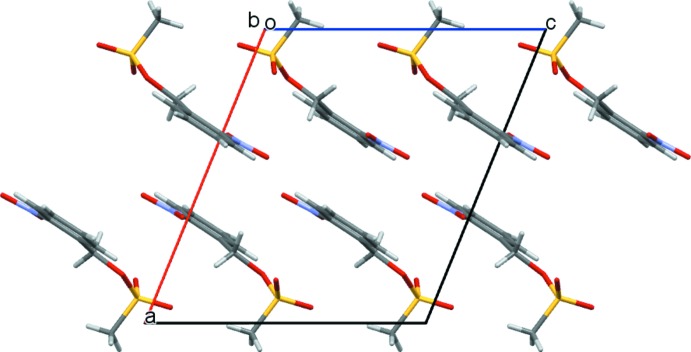
A viewed along the b axis of the crystal packing of the title compound.

## supplementary crystallographic information

### 1. Comment

Nitroalkenes have been used as substrates for Michael addition reactions (Ranu & Banerjee, 2005) and for the synthesis of many organic molecules (Ballini *et al.*, 2005).

The *ORTEP* of the title molecule is shown in figure 1. The molecules in the crystal structure are connected with C—H···O hydrogen bonds (Table 1). The C8—H8B···O4 hydrogen bond exhibits ring motifs of the type *R*^2^_2_(8). The overall geometry of the title compound is similar to the 3,5-dinitrobenzyl methanesulfonate (Khan *et al.*, 2008). Overall packing of the molecule is shown in figure 2.

### 2. Experimental

Red blocks were obtained from slow evaporation of a solution of ethanol.

### 3. Refinement

The hydrogen atom were fixed geometrically (C—H= 0.93–0.96 Å) and allowed to ride on their parent atoms with *U*_iso_(H) =1.5*U*_eq_(*C*-methyl) and = 1.2*U*_eq_(C).

### Crystal data

**Table:**

C_8_H_9_NO_5_S	*F*(000) = 480
*M**_r_* = 231.23	*D*_x_ = 1.526 Mg m^−^^3^
Monoclinic, *P*2_1_/*c*	Cu *K*α radiation, λ = 1.54178 Å
Hall symbol: -P 2ybc	Cell parameters from 1663 reflections
*a* = 12.414 (3) Å	θ = 3.8–64.5°
*b* = 7.967 (2) Å	µ = 2.94 mm^−^^1^
*c* = 10.994 (3) Å	*T* = 296 K
β = 112.235 (11)°	Block, red
*V* = 1006.5 (5) Å^3^	0.23 × 0.22 × 0.21 mm
*Z* = 4	

### Data collection

**Table:**

Bruker X8 Proteum CCD diffractometer	1663 independent reflections
Radiation source: Bruker MicroStar microfocus rotating anode	1541 reflections with *I* > 2σ(*I*)
Helios multilayer optics monochromator	*R*_int_ = 0.047
Detector resolution: 10.7 pixels mm^-1^	θ_max_ = 64.5°, θ_min_ = 3.9°
φ and ω scans	*h* = −14→13
Absorption correction: multi-scan (*SADABS*; Bruker, 2013)	*k* = −8→9
*T*_min_ = 0.552, *T*_max_ = 0.578	*l* = −12→9
6524 measured reflections	

### Refinement

**Table:**

Refinement on *F*^2^	Secondary atom site location: difference Fourier map
Least-squares matrix: full	Hydrogen site location: inferred from neighbouring sites
*R*[*F*^2^ > 2σ(*F*^2^)] = 0.045	H-atom parameters constrained
*wR*(*F*^2^) = 0.112	*w* = 1/[σ^2^(*F*_o_^2^) + (0.0554*P*)^2^ + 0.5912*P*] where *P* = (*F*_o_^2^ + 2*F*_c_^2^)/3
*S* = 1.08	(Δ/σ)_max_ < 0.001
1663 reflections	Δρ_max_ = 0.41 e Å^−^^3^
138 parameters	Δρ_min_ = −0.32 e Å^−^^3^
0 restraints	Extinction correction: *SHELXL97* (Sheldrick, 2008), FC^*^=KFC[1+0.001XFC^2^Λ^3^/SIN(2Θ)]^-1/4^
Primary atom site location: structure-invariant direct methods	Extinction coefficient: 0.0114 (9)

### Special details

**Table:**

Geometry. Bond distances, angles *etc*. have been calculated using the rounded fractional coordinates. All su's are estimated from the variances of the (full) variance-covariance matrix. The cell e.s.d.'s are taken into account in the estimation of distances, angles and torsion angles
Refinement. Refinement on *F*^2^ for ALL reflections except those flagged by the user for potential systematic errors. Weighted *R*-factors *wR* and all goodnesses of fit *S* are based on *F*^2^, conventional *R*-factors *R* are based on *F*, with *F* set to zero for negative *F*^2^. The observed criterion of *F*^2^ > σ(*F*^2^) is used only for calculating -*R*-factor-obs *etc*. and is not relevant to the choice of reflections for refinement. *R*-factors based on *F*^2^ are statistically about twice as large as those based on *F*, and *R*-factors based on ALL data will be even larger.

### Fractional atomic coordinates and isotropic or equivalent isotropic displacement parameters (Å^2^)

**Table:**

	*x*	*y*	*z*	*U*_iso_*/*U*_eq_	
S1	0.08637 (5)	0.25179 (7)	0.06271 (5)	0.0405 (2)	
O1	0.43500 (18)	0.3906 (3)	0.69206 (16)	0.0672 (7)	
O2	0.36003 (19)	0.2381 (2)	0.52088 (19)	0.0601 (7)	
O3	0.16548 (14)	0.3941 (2)	0.15092 (14)	0.0458 (5)	
O4	0.05351 (17)	0.3133 (3)	−0.06740 (15)	0.0592 (7)	
O5	0.14617 (16)	0.0958 (2)	0.09607 (18)	0.0584 (6)	
N1	0.38559 (15)	0.3744 (2)	0.57357 (17)	0.0376 (6)	
C1	0.35397 (16)	0.5255 (2)	0.49184 (19)	0.0295 (6)	
C2	0.3940 (2)	0.6755 (3)	0.5560 (2)	0.0411 (7)	
C3	0.3686 (2)	0.8231 (3)	0.4862 (3)	0.0523 (9)	
C4	0.3040 (2)	0.8185 (3)	0.3534 (3)	0.0533 (9)	
C5	0.2634 (2)	0.6678 (3)	0.2901 (2)	0.0424 (7)	
C6	0.28708 (17)	0.5157 (3)	0.35717 (19)	0.0310 (6)	
C7	0.2405 (2)	0.3532 (3)	0.2870 (2)	0.0387 (7)	
C8	−0.0341 (2)	0.2504 (3)	0.1059 (3)	0.0526 (9)	
H2	0.43780	0.67660	0.64580	0.0490*	
H3	0.39490	0.92490	0.52830	0.0630*	
H4	0.28730	0.91790	0.30560	0.0640*	
H5	0.21920	0.66820	0.20030	0.0510*	
H7A	0.30420	0.28170	0.28830	0.0460*	
H7B	0.19630	0.29430	0.32990	0.0460*	
H8A	−0.01050	0.22110	0.19700	0.0790*	
H8B	−0.08950	0.16960	0.05320	0.0790*	
H8C	−0.06920	0.35980	0.09150	0.0790*	

### Atomic displacement parameters (Å^2^)

**Table:**

	*U*^11^	*U*^22^	*U*^33^	*U*^12^	*U*^13^	*U*^23^
S1	0.0457 (4)	0.0446 (4)	0.0265 (3)	−0.0009 (2)	0.0083 (3)	−0.0091 (2)
O1	0.0821 (13)	0.0607 (13)	0.0332 (10)	0.0016 (10)	−0.0071 (9)	0.0119 (8)
O2	0.0803 (13)	0.0282 (9)	0.0564 (12)	0.0036 (8)	0.0083 (10)	0.0025 (7)
O3	0.0555 (10)	0.0432 (9)	0.0289 (8)	−0.0069 (7)	0.0050 (7)	−0.0037 (7)
O4	0.0670 (11)	0.0806 (14)	0.0262 (9)	−0.0037 (10)	0.0134 (8)	−0.0061 (8)
O5	0.0595 (11)	0.0463 (11)	0.0559 (11)	0.0051 (8)	0.0065 (9)	−0.0161 (8)
N1	0.0376 (9)	0.0337 (10)	0.0345 (10)	0.0023 (7)	0.0056 (8)	0.0053 (8)
C1	0.0322 (10)	0.0256 (10)	0.0298 (10)	0.0008 (8)	0.0106 (8)	0.0013 (8)
C2	0.0463 (12)	0.0365 (12)	0.0349 (12)	−0.0059 (10)	0.0090 (10)	−0.0070 (9)
C3	0.0668 (16)	0.0272 (12)	0.0591 (16)	−0.0085 (11)	0.0197 (13)	−0.0070 (11)
C4	0.0712 (16)	0.0295 (12)	0.0571 (16)	−0.0019 (11)	0.0220 (13)	0.0121 (11)
C5	0.0531 (13)	0.0382 (13)	0.0325 (11)	−0.0006 (10)	0.0123 (10)	0.0058 (9)
C6	0.0339 (10)	0.0299 (11)	0.0303 (10)	−0.0002 (8)	0.0135 (9)	−0.0014 (8)
C7	0.0465 (12)	0.0361 (12)	0.0279 (11)	−0.0004 (9)	0.0079 (9)	−0.0034 (8)
C8	0.0529 (15)	0.0635 (18)	0.0402 (14)	−0.0060 (11)	0.0163 (12)	−0.0060 (11)

### Geometric parameters (Å, º)

**Table:**

S1—O3	1.5715 (17)	C4—C5	1.384 (3)
S1—O4	1.4181 (18)	C5—C6	1.391 (3)
S1—O5	1.4227 (18)	C6—C7	1.506 (3)
S1—C8	1.732 (3)	C2—H2	0.9300
O1—N1	1.219 (2)	C3—H3	0.9300
O2—N1	1.215 (2)	C4—H4	0.9300
O3—C7	1.469 (3)	C5—H5	0.9300
N1—C1	1.464 (2)	C7—H7A	0.9700
C1—C2	1.381 (3)	C7—H7B	0.9700
C1—C6	1.399 (3)	C8—H8A	0.9600
C2—C3	1.374 (3)	C8—H8B	0.9600
C3—C4	1.375 (4)	C8—H8C	0.9600
			
O3—S1—O4	104.31 (11)	O3—C7—C6	107.68 (18)
O3—S1—O5	109.14 (10)	C1—C2—H2	120.00
O3—S1—C8	103.78 (11)	C3—C2—H2	120.00
O4—S1—O5	119.00 (13)	C2—C3—H3	120.00
O4—S1—C8	109.28 (14)	C4—C3—H3	120.00
O5—S1—C8	110.13 (12)	C3—C4—H4	120.00
S1—O3—C7	118.41 (14)	C5—C4—H4	120.00
O1—N1—O2	122.6 (2)	C4—C5—H5	119.00
O1—N1—C1	118.59 (18)	C6—C5—H5	119.00
O2—N1—C1	118.77 (17)	O3—C7—H7A	110.00
N1—C1—C2	115.91 (17)	O3—C7—H7B	110.00
N1—C1—C6	121.17 (17)	C6—C7—H7A	110.00
C2—C1—C6	122.93 (18)	C6—C7—H7B	110.00
C1—C2—C3	119.6 (2)	H7A—C7—H7B	108.00
C2—C3—C4	119.3 (2)	S1—C8—H8A	109.00
C3—C4—C5	120.8 (2)	S1—C8—H8B	110.00
C4—C5—C6	121.7 (2)	S1—C8—H8C	110.00
C1—C6—C5	115.73 (19)	H8A—C8—H8B	109.00
C1—C6—C7	123.31 (19)	H8A—C8—H8C	109.00
C5—C6—C7	120.95 (18)	H8B—C8—H8C	109.00
			
O4—S1—O3—C7	−163.22 (18)	C2—C1—C6—C5	0.5 (3)
O5—S1—O3—C7	−35.1 (2)	C2—C1—C6—C7	−178.4 (2)
C8—S1—O3—C7	82.36 (19)	N1—C1—C6—C7	1.5 (3)
S1—O3—C7—C6	−168.44 (15)	C1—C2—C3—C4	−0.2 (4)
O1—N1—C1—C2	6.4 (3)	C2—C3—C4—C5	0.8 (4)
O2—N1—C1—C2	−174.7 (2)	C3—C4—C5—C6	−0.7 (4)
O2—N1—C1—C6	5.4 (3)	C4—C5—C6—C1	0.1 (4)
O1—N1—C1—C6	−173.6 (2)	C4—C5—C6—C7	179.0 (2)
C6—C1—C2—C3	−0.4 (4)	C1—C6—C7—O3	174.4 (2)
N1—C1—C6—C5	−179.6 (2)	C5—C6—C7—O3	−4.4 (3)
N1—C1—C2—C3	179.7 (2)		

### Hydrogen-bond geometry (Å, º)

**Table:**

*D*—H···*A*	*D*—H	H···*A*	*D*···*A*	*D*—H···*A*
C2—H2···O1^i^	0.93	2.54	3.266 (3)	135
C3—H3···O2^ii^	0.93	2.53	3.335 (3)	145
C7—H7*B*···O4^iii^	0.97	2.58	3.539 (3)	169
C8—H8*A*···O4^iii^	0.96	2.42	3.374 (4)	172

Symmetry codes: (i) −*x*+1, *y*+1/2, −*z*+3/2; (ii) *x*, *y*+1, *z*; (iii) *x*, −*y*+1/2, *z*+1/2.
